# A Bayesian incorporated linear non-Gaussian acyclic model for multiple directed graph estimation to study brain emotion circuit development in adolescence

**DOI:** 10.1162/netn_a_00384

**Published:** 2024-10-01

**Authors:** Aiying Zhang, Gemeng Zhang, Biao Cai, Tony W. Wilson, Julia M. Stephen, Vince D. Calhoun, Yu-Ping Wang

**Affiliations:** School of Data Science, University of Virginia, Charlottesville, VA, USA; Department of Biomedical Engineering, Tulane University, New Orleans, LA, USA; Institute for Human Neuroscience, Boys Town National Research Hospital, Boys Town, NE, USA; Mind Research Network, Albuquerque, NM, USA; Tri-institutional Center for Translational Research in Neuroimaging and Data Science (TReNDS), Georgia State University, Georgia Institute of Technology, Emory University, Atlanta, GA, USA

**Keywords:** Adolescence, Brain development, fMRI, Emotion processing, Bayesian network

## Abstract

Emotion perception is essential to affective and cognitive development which involves distributed brain circuits. Emotion identification skills emerge in infancy and continue to develop throughout childhood and adolescence. Understanding the development of the brain’s emotion circuitry may help us explain the emotional changes during adolescence. In this work, we aim to deepen our understanding of emotion-related functional connectivity (FC) from association to causation. We proposed a Bayesian incorporated linear non-Gaussian acyclic model (BiLiNGAM), which incorporated association model into the estimation pipeline. Simulation results indicated stable and accurate performance over various settings, especially when the sample size was small. We used fMRI data from the Philadelphia Neurodevelopmental Cohort (PNC) to validate the approach. It included 855 individuals aged 8–22 years who were divided into five different adolescent stages. Our network analysis revealed the development of emotion-related intra- and intermodular connectivity and pinpointed several emotion-related hubs. We further categorized the hubs into two types: in-hubs and out-hubs, as the center of receiving and distributing information, respectively. In addition, several unique developmental hub structures and group-specific patterns were discovered. Our findings help provide a directed FC template of brain network organization underlying emotion processing during adolescence.

## Author Summary

Our study introduces a novel method for analyzing directed graphs across multiple groups and demonstrates its effectiveness through a series of simulation studies. This method is applied to investigate the development of directed functional connectivity for emotion processing across diverse adolescent periods. Our findings unveil a notable increase in interfunctional connectivity with age, specifically involved with the executive control and memory retrieval, indicating the maturation of emotion processing function. Additionally, significant development of intraconnectivity in the subcortical areas emerges in early adolescence, whereas development of cerebellum emerges in the very end of adolescence. These insights offer valuable contributions to our understanding of the dynamic neural processes underlying emotion regulation during adolescence.

## INTRODUCTION

Emotion perception is essential to affective and cognitive development, which plays a pivotal role in social interactions and mental health, especially during adolescence—a critical period characterized by significant emotional and cognitive transformations. The ability to identify distinct facial expressions is fundamental for recognizing others’ emotional states and is supported by distributed brain circuits that continue to develop throughout childhood and adolescence (Gee et al., 2022; Zhang, Padmanabhan, Gross, & Menon, 2019). Understanding the development of the brain’s emotion circuits during this period can shed light on the process of emotional maturation in adolescents and offer insights into potential neural deviations that may indicate a risk for psychiatric disorders.

Functional magnetic resonance imaging (fMRI) has been widely applied to study functional development in emotion circuits, because of its relative simplicity of use, noninvasive nature, and relatively high spatial resolution. In our previous work (Zhang, Cai, et al., 2020), we studied the fMRI images collected from the Philadelphia Neurodevelopmental Cohort (PNC) and delineated the trajectory of brain functional connectivity (FC) from late childhood (preadolescence) to early adulthood (postadolescence) during emotion identification tasks. The FC metrics that we used were defined by statistical associations (partial correlations, in particular) between measured brain regions. However, it has been pointed out that the statistical association may be problematic in that it only reveals the spatial connections but not causal information (Reid et al., 2019). Approaches that characterize statistical associations are likely a good starting point, but the directionality of brain connections (i.e., directed functional connectivity [dFC]) should provide more informative insights.

Directed acyclic graph (DAG) models, also known as Bayesian networks, are designed to model causal relationships in complex systems. Many fMRI studies have utilized the DAG models to characterize the dFC and elucidate their differences between different population groups (Henry & Gates, 2017; Smith et al., 2011). Iyer et al. (2013) validated the Peter Spirtes and Clark Glymour algorithm (PC algorithm, Spirtes et al. (2000)) in dFC estimation; Hanson, Hanson, Ramsey, and Glymour (2013) applied the greedy equivalent search (GES) method (Chickering, 2002) and investigated the differences in brain integration between neurotypical controls and patients with autism spectrum disorders; Liu et al. (2015) employed the linear non-Gaussian acyclic models (LiNGAM) (Shimizu, Hoyer, Hyvärinen & Kerminen, 2006) to account for the non-Gaussianity of fMRI data and revealed network differences between patients with bipolar disorders and those with major depression; Zhang et al. (2022) improved the Non-combinatorial Optimization via Trace Exponential and Augmented lagRangian for Structure learning (Notears, Zheng, Aragam, Ravikumar, and Xing (2018)) to detect abnormal dFC in schizophrenia.

Despite the success of DAGs in case-control comparisons, many biomedical applications involve data from multiple groups. Our study, for instance, aims to estimate multiple DAGs to reveal the development of emotion-related dFC across different adolescent periods. This raises an important statistical question, namely how to jointly estimate related graphical models in order to effectively make use of the available data (Wang, Segarra, & Uhler, 2018). Oates, Smith, Mukherjee, and Cussens (2016) presented an ILP approach for joint estimation over multiple DAGs for small graphs. Later, Wang et al. (2018) proposed jointGES for Gaussian distributed data under high-dimensional cases. Specifically for non-Gaussian distributed fMRI data (Ramsey, Sanchez-Romero, & Glymour, 2014), Shimizu (2012) developed a joint estimation method for multiple LiNGAMs; Ramsey et al. (2010) proposed the independent multisample GES (IMaGES) algorithm to account for graphical differences and proved usefulness in understanding emotion regulation during the child-to-adolescent development (Gee et al., 2022). However, most methods assume that the DAGs across groups share the same directed connectivity structure while the connection strengths can vary (Ramsey, Hanson, & Glymour, 2011; Shimizu, 2012), which is not always true in reality. To relax the common-structure limitation, we propose the Bayesian incorporated linear non-Gaussian acyclic model (BiLiNGAM). The BiLiNGAM leverages insights from the association networks as prior information to narrow down the set of plausible DAG spaces. Specifically, we implement a joint Bayesian estimation approach (Zhang, Cai, et al., 2020) to acquiring the priors for multiple groups, optimizing for group similarities while accommodating their unique characteristics. A series of simulation studies have been conducted to illustrate the advantages of BiLiNGAM in terms of convergence speed and accuracy, especially in high dimensional datasets.

We applied our proposed model to fMRI data collected during an emotion identification task from participants in the Philadelphia Neurodevelopmental Cohort (PNC). Our analysis aimed to discern emotion-related dFC patterns across five distinct adolescent groups, shedding light on the nuanced changes within the emotion circuitry during this critical developmental period. By examining both common and distinctive dFC patterns, we delved into the evolving trajectories of functional network modules and identified pivotal hub regions of interest (ROIs) in the adolescent brain.

## METHODS AND MATERIALS

In this section, we first introduce the proposed BiLiNGAM model, specifically designed for estimating multiple DAGs that are distinct yet related, particularly in high-dimensional settings. Then we describe the simulation studies to demonstrate the advantages of BiLiNGAM model and the application to investigate emotion-related dFC patterns across different adolescence periods.

### Background: DAG and LiNGAM

A directed acyclic graph (DAG) is a probabilistic graphical model that represents a set of variables and their conditional dependencies via the directed edges. Formally, a DAG is denoted by *G* = (*V*, *E*) where *V* = {1, 2, …, *p*} represents the set of nodes and *E* ⊂ *V* × *V* represents the set of directed edges. The nodes in set *V* correspond to the set of variables **X** = (*X*_1_, *X*_2_, …, *X*_*p*_)^*T*^, which are ROIs in our case. An edge (*i*, *j*) is directed if (*i*, *j*) ∈ *E* but (*j*, *i*) ∉ *E*, and we denote it as *i* → *j*. A DAG is acyclic, meaning that there are no circular dependencies within the graph.

The linear non-Gaussian acyclic model (LiNGAM) used the structural equation model (SEM) to estimate the DAG for non-Gaussian variables (Shimizu et al., 2006). Let **B** = {*b*_*ij*_} ∈ *R*^*p*×*p*^ be the weighted adjacency matrix specifying the edge weights of the underlying DAG *G*. The observed random vector **X** = (**X**_1_, **X**_2_, …, **X***_p_*) ∈ *R*^*p*^ is assumed to be generated from the following linear SEM,

$$X=B^{T}X+\epsilon ,$$

where ***ϵ*** = (***ϵ***_1_, ***ϵ***_2_, …, ***ϵ***_*p*_) is a continuous random vector; the ***ϵ***_*i*_’s, ∀*i* = 1, 2, …, *p* have non-Gaussian distributions with nonzero variances, and are independent of each other. A property of acyclicity is that there exists at least one permutation *π* of *p* variables such that *b*_*ij*_ = 0, ∀*π*(*i*) < *π*(*j*). In other words, the weight matrix **B** can be reordered to a strictly lower triangular matrix according to the permutation *π*. The goal of LiNGAM is to find the correct permutation and estimate the weight matrix **B**. Since the components of ***ϵ*** are independent and non-Gaussian, Shimizu et al. (2006) first proposed the independent component analysis (ICA) based algorithm known as ICA-LiNGAM. Later, the direct LiNGAM (Shimizu et al., 2011) was developed, which estimates the causal order of variables by successively subtracting the effect of each independent component from the given data. Compared to the ICA-based algorithm, the direct LiNGAM needs no initial guess or algorithmic parameters and has guaranteed convergence.

In fMRI studies, it has been demonstrated that the non-Gaussianity can provide valuable orientation information, leading to more accurate identification of directed graph structures compared to traditional Gaussian settings (Ramsey et al., 2014). However, when dealing with heterogeneous data with limited sample size, the LiNGAMs may not yield satisfactory estimations or fail under high dimensional setting. This highlights the need for additional modeling techniques to address these challenges and bridge the gap in application.

### Bayesian Incorporating Priors

#### Incorporating association networks as prior information.

To overcome the sample size limitation, we propose to incorporate prior knowledge to direct LiNGAM, thereby reducing the searching space and thus largely accelerating the convergence rate to true graph and improving the computation efficiency. Reid et al. (2019) has highlighted that generalizing insights from the FC studies measured by statistical associations can facilitate the inference of conditional dependency. Current FC methods mainly employ three types of association measures, namely, the Pearson correlation (Rasero et al., 2023), partial correlation (Zhang, Cai, et al., 2020), and distance correlation (Xiao et al., 2022). Pearson correlation describes the linear correlation of a pair of variables, partial correlation measures the association between two variables removing the effect of other variables, and distance correlation can reflect nonlinear associations between two variables. Within a linear model, a partial correlation-based approach is more appropriate to incorporate as the prior than the Pearson correlation. This is because in a complex system like the brain network, the Pearson correlation is much weaker marginally (Liang, Song, & Qiu, 2015), that is, all nodes (variables) are directly or indirectly correlated, making it difficult to distinguish significant connections from a dense network. On the contrary, partial correlations can explore direct associations between two nodes, controlling for the confounding variables, which can facilitate the inference of influential directions. In our previous study (Zhang, Zhang, et al., 2020), we incorporated the partial correlation network using the *ψ*-learning method (Liang et al., 2015) as a prior with LiNGAM and showed its superiority in both of convergence and accuracy.

#### Bayesian joint prior estimation for multiple groups.

We proposed the joint Bayesian-incorporating *ψ*-learning to address heterogeneity across multiple groups, which consisted of three steps: Step 1, Gaussian transformation; Step 2, distinct and common graph construction; and Step 3, prior matrices acquisition. Many partial correlation–based methods are designed for Gaussian distributed data due to their mathematical simplicity (Zhang, Fang, Liang, Calhoun, & Wang, 2019). Liu, Lafferty, and Wasserman (2009) have proposed a nonparanormal transformation, which relaxes the Gaussian assumption to any continuous one, which is therefore applied in Step 1. For Step 2, we consider both the distinct and common structure in each group. The distinct graph estimation for each group is implemented through the *ψ*-learning method as in Zhang, Fang, et al. (2019). To strengthen the similarities over various groups, we adopt the same Bayesian incorporating joint estimation method as in our previous study (Zhang, Cai, et al., 2020). The similarities are highlighted through proper Bayesian priors and a meta-analysis procedure. Finally, we use the union of the distinct estimated graph and joint estimated graph as the prior for each group.

### BiLiNGAM

We now illustrate the proposed BiLiNGAM to jointly estimate multiple DAGs for non-Gaussian data. First, we apply the joint Bayesian-incorporated *ψ*-learning to acquire FC networks as the prior information. For each group *k*, *k* = 1, …, *K*, we obtain its distinctly estimated edge structure *E*^*d*,*k*^ through *ψ*-learning and its jointly estimated edge structure with strengthened group similarities *E*_*c*,*k*_ through the Bayesian incorporating joint estimation from the observations **X**^*k*^ ∈ *R*^*n*_*k*_×*p*^, where *n*_*k*_ represents the number of samples in group *k* and *p* represents the number of variables. We then introduce the prior matrix *A*^*prior*^ to represent the potential directed edge space, where a value of 0 indicates no directed edge, and −1 indicates uncertainty about the edge status. The prior matrix **A**^*prior*,*k*^ = {$a_{ij}^{\text{prior},k}$} of group *k* combines the edge structures from *E*^*d*,*k*^ and *E*_*c*,*k*_, which is defined as

$$a_{ij}^{\text{prior},k}≔\left\{\begin{array}{l} 0,\left(i,j\right)\notin E^{d,k}\cup E_{c,k}\\ -1,\text{otherwise}. \end{array}\right.$$

Next we leverage the prior matrix **A**^*prior*,*k*^ with the direct LiNGAM to estimate the weighted adjacency matrix of the DAG for group *k*, that is, **B**^*k*^. The detailed procedure is summarized in Algorithm 1 with code available at https://github.com/Aiying0512/BiLiNGAM.

**Algorithm 1.** BiLiNGAM algorithm

**Input:** Collection of observation **X**^*k*^ = ($X_{i}^{k}$) ∈ ℝ^*n*_*k*_×*p*^, where *k* = 1, 2, …, *K*, *i* = 1, 2, …, *p* and $X_{i}^{k}$’s are non-Gaussian continuous.
**Output:** Collection of estimated weighted adjacency matrices $\hat{B}$^*k*^
1. Prior estimation: joint Bayesian-incorporating *ψ*-learning.
**Start:**
a. For *k* = 1, 2, …, *K*, use the nonparanormal transformation (Liu et al., 2009) to render **X**^*k*^ normal (Gaussian).
b. Apply the *ψ*-learning method (Liang et al., 2015) to each group *k*, *k* = 1, 2, …, *K* separately for distinct estimation and acquire the adjacency matrix **E**^*d*,*k*^.
c. Apply the Bayesian incorporating joint estimation (Zhang, Padmanabhan, et al., 2019) to strengthen the similarities among the groups and acquire the **E**^*c*,*k*^, ∀*k*.
d. Extract the prior matrix **A**^*prior*,*k*^ from **E**^*prior*,*k*^ = **E**^*c*,*k*^ ∪ **E**^*d*,*k*^ where $a_{ij}^{\text{prior},k}$ = −1 if $e_{ij}^{\text{prior},k}$ = 1 otherwise $a_{ij}^{\text{prior},k}$ = 0.
**End**
2. Obtain the estimated weighted DAG adjacency matrices $\hat{B}$^*k*^: LiNGAM.
**Start:** For each *k*
a. Identify the casual order *π*^*k*^ using the direct LiNGAM with the prior matrix **A**^*prior*,*k*^ (Shimizu et al., 2011).
b. Construct a strictly lower triangular matrix $\tilde{B}$^*k*^ by following the order *π*^*k*^, and the corresponding $\tilde{A}$^*prior*,*k*^ with the same order.
c. Estimate the connection strengths (${\tilde{B}}_{j}^{k}$)^*T*^ = (${\tilde{b}}_{1j}^{k}$, ${\tilde{b}}_{2j}^{k}$, …, ${\tilde{b}}_{pj}^{k}$) consistent with $\tilde{A}$^*prior*,*k*^ by solving sparse regressions of the form $${\hat{\tilde{B}}}_{j}^{k}=\arg\min_{{\tilde{B}}_{j}^{k}\subset \mathrm{supp}\left({\tilde{a}}_{j}^{\text{prior},k}\right)}{\left\|X_{j}^{k}-X^{k}{\tilde{B}}_{j}^{k}\right\|}_{2}^{2}$$
d. Obtain $\hat{B}$^*k*^ by converting $\tilde{B}$^*k*^ to the original order.
**End**

### Simulation Data Generation

We evaluated the performance of the joint estimation of *K* different DAGs where we varied *K* ∈ {3, 5}. For all experiments, we set the number of nodes *p* = 200 and the total number of observations *N* = 750. For each group, we set the number of samples equally, that is, *n*_1_ = *n*_2_ = … = *n*_*K*_ = *n* = *N*/*K*. The random DAG *G* is simulated through the R package *pcalg*, and density of the graph is controlled by the edge probability *d*/(*p* − 1), where *d* is the mean edge degree parameter with values {1, 2, 5}. The true DAGs generation procedure is illustrated as follows. We first used the *pcalg*, to generate *G*^1^. Given *G*^1^, we assigned uniformly random weights to the edges to obtain the weighted adjacency matrix **B**^1^ = ($b_{ij}^{1}$): $b_{ij}^{1}$ ∼ Unif(−0.8, −0.3) ∪ (0.3, 0.8), if there is an edge *i* → *j*, otherwise $b_{ij}^{1}$ = 0. For *G*^*k*^, *k* = 2, 3, …, *K*, we followed the same random edge deleting-adding procedure in a sequential manner. We randomly removed 5% edges in *G*^*k*−1^, *k* = 2, …, 5, by setting the corresponding nonzero elements in **B**^*k*^ to be 0, and then added 5% edges at random by giving them values drawn from the uniform distribution *U*[0.3, 0.5] to obtain **B**^*k*^. Given **B**^*k*^’s, we generated **X**^*k*^ = (**B**^*k*^)^*T*^**X**^*k*^ + ***ϵ***^*k*^ ∈ *R*^*p*^ with ***ϵ***^*k*^ from chi-squared (Chisq) noise with degree of freedom 1 and zero mean, that is, $\epsilon_{i}^{k}$ ∼ $\chi_{1}^{2}$ − 1, *i*, = 1, 2, …, *p*, *k* = 1, 2, …, *K*.

We considered four methods for comparison, which are the PC algorithm (Spirtes et al., 2000), the IMaGES (Ramsey et al., 2010), the ICA-LiNGAM (Shimizu et al., 2006), and the *ψ*-LiNGAM that we proposed previously (Zhang, Zhang, et al., 2020). The PC and ICA-LiNGAM were implemented through the R package *pcalg*, and the IMaGES was implemented through the *Tetrad* toolbox. The codes for *ψ*-LiNGAM and BiLiNGAM are available at https://github.com/Aiying0512. We set the significance level *α* = 0.05 with FDR correction for the PC, *ψ*-LiNGAM and BiLiNGAM.

For each scenario, 10 datasets were simulated independently. We assessed the performances of the four methods through the true positive rate (TPR), false discovery rate (FDR), and structural hamming distance (SHD) (Tsamardinos, Brown, & Aliferis, 2006). TPR and FDR are two common measures of binary classification. Let us define an experiment from *P* positive instances and *N* negative instances for some conditions. In our case, the positive instance represents a directed edge from one node to the other. The four outcomes are summarized in Table 1. The definitions of TPR and FDR are given as follows:

$$\text{TPR}=\frac{\text{TP}}{\text{TP}+\text{FN}},\;\text{FDR}=\frac{\text{FP}}{\text{FP}+\text{TP}}.$$

SHD is a frequently used metric based on the number of operations needed to transform the estimated DAG into the true graph (Kalisch & Bühlmann, 2007). In simple terms, SHD counts the total number of edge insertions, deletions, or flips during the transformation.

**Table 1.** Outcomes of a binary decision

	Actual positive (*P*)	Actual negative (*N*)
Predicted positive	True positive (TP)	False positive (FP)
Predicted negative	False negative (FN)	True negative (TN)

### Analyses on the PNC Dataset

The Philadelphia Neurodevelopmental Cohort (PNC) dataset consists of fMRI images under emotion identification task from 855 individuals. The age range of the participating subjects was between 8 and 22 years. Due to physical and cognitive changes (Zhang, Cai, et al., 2020), we divided them into five groups, each representing a period related to adolescence (see Table 2).

**Table 2.** Group division information

Category index	Group name	Age range	Number of subjects
1	Preadolescence	8–12	194
2	Early adolescence	12–14	150
3	Middle adolescence	14–16	158
4	Late adolescence	16–18	166
5	Postadolescence	18–22	187

#### Imaging acquisition and preprocessing.

**Figure 1.**
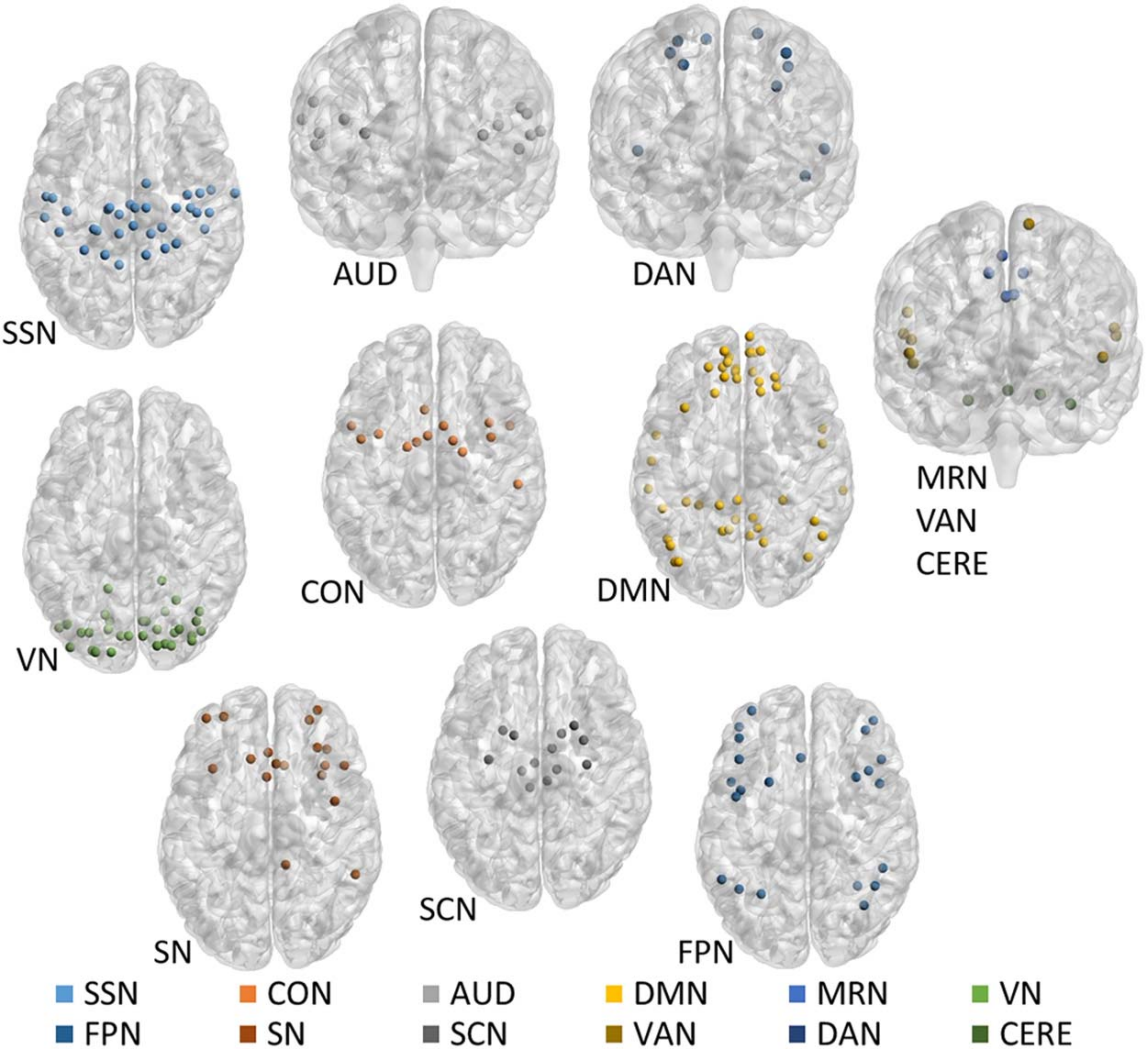
Twelve functional network modules based on the 264 nodes from the template defined by Power et al. (2010).

All MRI scans were acquired on a single 3T Siemens TIM Trio whole-body scanner. During the task, each subject was asked to label emotions displayed, which include happy, angry, sad, fearful, and neutral faces. The total scan duration was 10.5 min. Blood oxygenation level–dependent (BOLD) fMRI was acquired using a whole-brain, single-shot, multislice, gradient-echo (GE) echoplanar (EPI) sequence of 124 volumes (372 s) with the following parameters TR/TE = 3, 000/32 ms, flip = 90°, FOV = 192 × 192 mm, matrix = 64 × 64, slice thickness/gap = 3 mm/0 mm. The resulting nominal voxel size was 3.0 × 3.0 × 3.0 mm (Satterthwaite et al., 2014). Standard preprocessing steps were applied using SPM12, including motion correction, spatial normalization to standard MNI space, and spatial smoothing with a 3-mm full width at half max (FWHM) Gaussian kernel. Then multiple regression considering the influence of motion was performed and the stimulus on-off contrast maps for each subject were obtained. Finally, 264 functionally defined regions of interest (ROIs) were extracted based on the power template (Power, Fair, Schlaggar, & Petersen, 2010). The 264 ROIs (nodes) can be divided into 12 functional network (FN) modules as provided by Power et al. (2010) (see Figure 1), including sensory/somatomotor network (SSN), cingulo-opercular task control network (CON), auditory network (AUD), default mode network (DMN), memory retrieval network (MRN), visual network (VN), fronto-parietal task control network (FPN), salience network (SN), subcortical network (SCN), ventral attention network (VAN), dorsal attention network (DAN), and cerebellum network (CERE).

#### Analytical pipeline to understand emotion circuit development.

**Figure 2.**
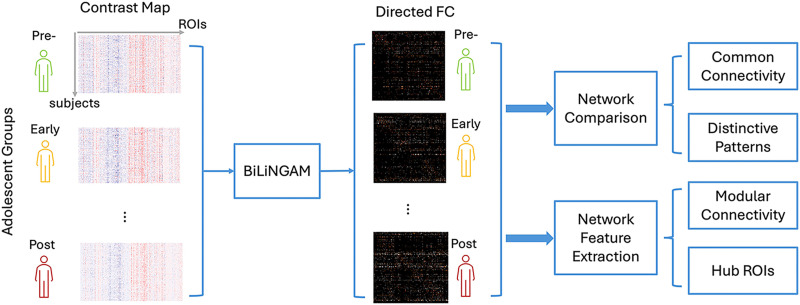
Schematic illustration of the analysis pipeline with the proposed BiLiNGAM model.

As shown in Figure 2, we investigate the development of emotion-related dFC from two aspects: (1) comparing the common and distinctive dFC patterns; (2) extracting significant network features in terms of functional network modules and hub ROIs. The intra- and intermodular connectivity is calculated by counting the number of edges module-wise. A hub ROI refers to the region with a large number of edges, or a high degree, in the brain network. Here we define hubs as the nodes with degrees at least two standard deviations higher than the average degrees within the DAG (Fang et al., 2017). For a DAG, the degree of a node is composed of two elements: in-degree and out-degree, which are named according to the direction of the edges. Based on the types of degrees, we identified the hub structures: in-hub as the center to receive information, and out-hub as the center to send information.

## RESULTS

### Simulation Results

**Figure 3.**
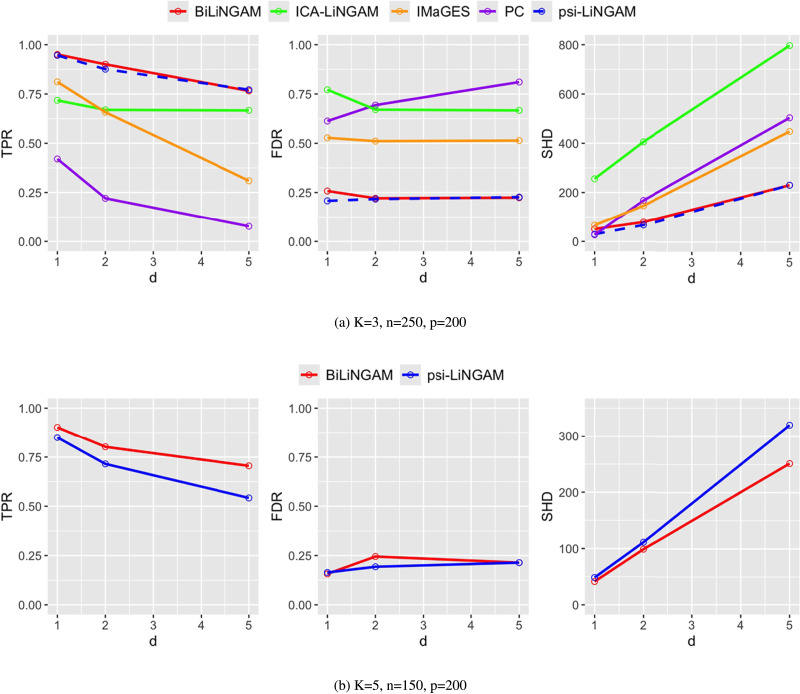
Simulation results using various estimation methods with different network densities by varying the mean edge degree parameter *d* ∈ {1, 2, 5}. The average performance in terms of TPR (left), FDR (middle) and SHD (right) is presented for the setting of *N* = 750 total observations for *K* groups with *p* = 200 variables, where the number of groups *K* ∈ {3, 5} with equal observations in each group *n* = *N*/*K*.

Figure 3A gives the results for *K* = 3, where each group has *n* = 250 samples and *p* = 200 nodes. We compared the results of all five methods with various graph densities controlled by the mean edge degree parameter *d*. The simulation results reveal the limited edge orientation ability of the PC and IMaGES. While the TPR curve of ICA-LiNGAM demonstrates reasonable performance, the high FDR and SHD values indicate that ICA-LiNGAM requires a larger number of observations to converge to the true graph. Both methods incorporating association networks as prior information (*ψ*-LiNGAM and BiLiNGAM) exhibit similarly strong performance. This suggests that when the sample size is sufficiently large (*n* > *p*), incorporating associations into LiNGAM models can significantly improve causal inferences.

Further, we compared the two methods under high-dimensional cases (i.e., *n* < *p*). Figure 3B shows the results for *K* = 5, where each group has *n* = 150 samples. The FDR curves of the two methods maintain at the same low level. The TPR of BiLiNGAM is always higher than that of *ψ*-LiNGAM, while the SHD performs the opposite. In addition, as the mean edge degree parameter *d* increases, the differences in TPR and SHD also increase. Therefore, under high dimensional settings, both methods remain at a low FDR level, but BiLiNGAM outperforms *ψ*-LiNGAM in terms of TPR and SHD.

Overall, BiLiNGAM has maintained a stable and accurate performance over various settings. Specifically, under high-dimensional cases, the performance of BiLiNGAM is superior. When the sample size is adequate (i.e., *n* > *p*), BiLiNGAM performs at least as good as *ψ*-LiNGAM. More comprehensive simulation studies are presented in the Supporting Information section A.

### fMRI Results to Reveal the Emotion Circuit Development

We first conducted the Darling-Anderson test for non-Gaussianity and then applied BiLiNGAM. The parameter settings remain the same as in the simulation studies.

#### Common and distinctive dFC patterns across the groups.

**Figure 4.**
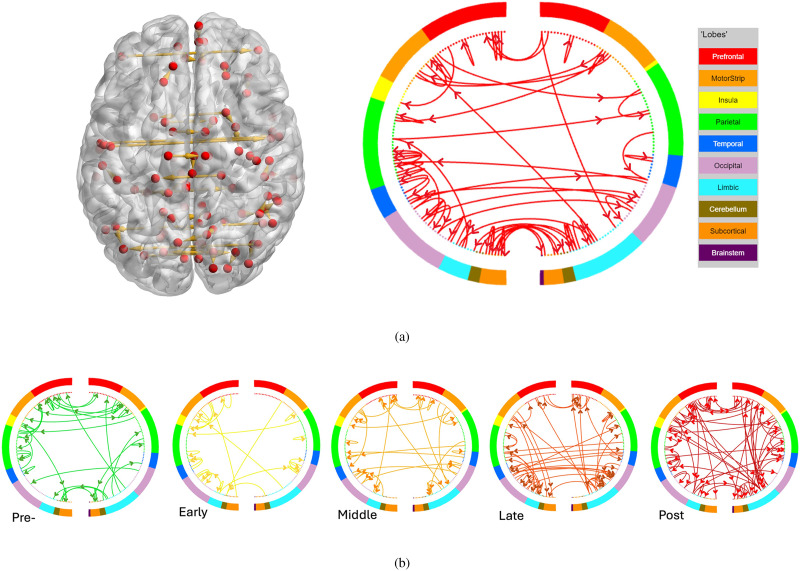
The common (A) and distinctive (B) dFC patterns of the five adolescent stages, where the arrows indicate the direction of the FC’s. (A) Left: the common directed FC in the axial view of the brain; Right: the common directed FC in network view, where the nodes are sorted by the lobes and separated into left (left half-circle) and right (right half-circle) hemispheres. (B) The distinctive dFC patterns of each adolescent groups in network view.

We extracted the common and distinctive directed connectivity patterns over the five groups as shown in Figure 4. As the brain develops, there is an increase in connections between the limbic system, responsible for emotion processing, and the prefrontal cortex, involved in higher order cognitive functions. Additionally, bilateral connections in the parietal and occipital lobes become more pronounced, supporting enhanced sensory integration and spatial awareness. These changes in connectivity facilitate the identification and regulation of emotions as part of the brain’s maturation process.

#### Development of emotion-related intra- and intermodular connectivity.

**Figure 5.**
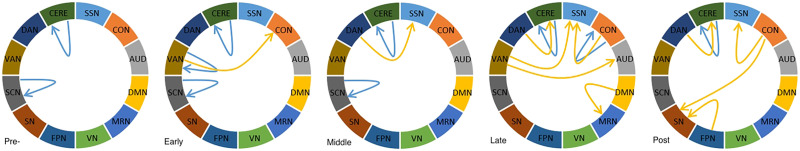
The development of modular network connectivity from preadolescence to postadolescence. The significant intra- and intermodule directed flows are visualized with blue and yellow arrows, respectively.

We examined intramodular and intermodular connectivity over the five adolescent groups. In the Supporting Information section B, we visualized the average directed edge degrees within and across modules, where the rows indicate the beginning of the arrows and the columns indicate the end of the arrows. From the heat maps, the intramodular connectivity of DMN, SCN, and CERE is strongly activated for all five groups. As age increases, there is increasing intermodular connectivity. We then conducted hypergeometric tests based on the number of edges module-wise and the significant intra- and interdirected connections are shown in Figure 5 at significance level *α* = 0.05 with FDR correction. The intramodular connectivity of the CERE was significantly activated of all adolescent groups for the emotion identification task, while the role of the SCN was only significant until the middle adolescent period. In addition, we found substantial intramodular connectivity of SCN in the early adolescent group and CON in the late adolescent group. From the aspect of intermodular connectivity, no significant directed flows were found in the preadolescent group, one each was identified for the early (VAN → CON) and middle (DAN → SSN) adolescent groups, five were identified for the late adolescent group (CON → SSN, VAN → SSN, VAN → AUD, DMN → MRN, DAN → CERE), and four directed flows were discovered in the postadolescent group (CON → SSN, DMN → MRN, CON → SN, FPN → SN).

#### Development of emotion-related hubs.

**Figure 6.**
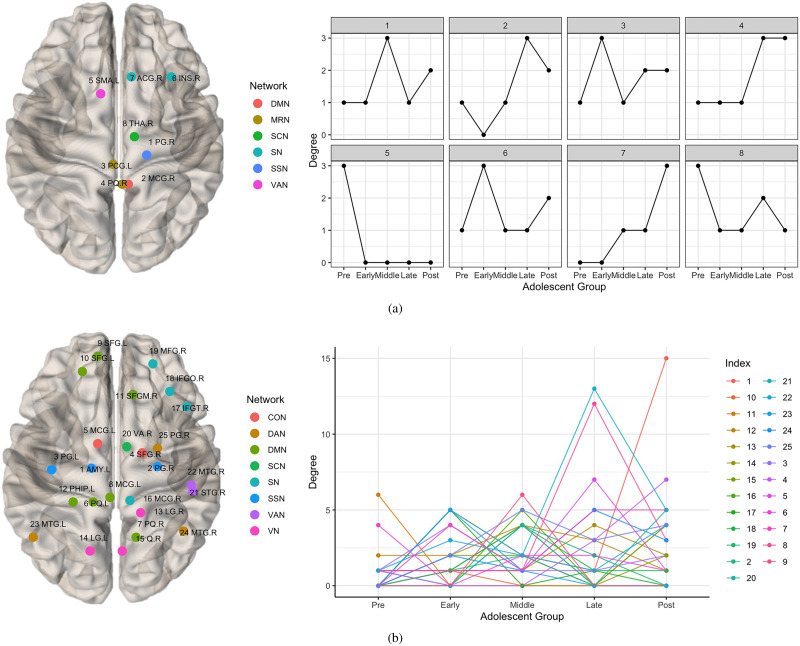
The developing trajectories of the identified inhubs (A) and outhubs (B), where the locations of the ROIs in the brain are visualized in the left. The color map of the ROIs represent the functional networks. The abbreviated name of the ROIs are provided and the detailed information can be found in the Supporting Information section B. The index of developing trajectories on the right correspond to the label on the left.

To gain more insights into the affective emotion circuits and their development with age, we analyzed hub nodes for each group. Figure 6A gives the in-hub development and the detailed in-hub information. The ROI at SMA.L has a preadolescence specific pattern. The ROIs at PQ.R and ACG.R have increased activities of receiving messages, especially, the ROI at ACG.R only starts to develop from middle adolescence. The remaining in-hubs have fluctuating trajectories. From Figure 6B, several out-hubs start to develop in a fluctuating manner after preadolescence, whose anatomical locations are at AMY.L, SFG.L (ROI 9, 10), PG.R, PQ.R, LG.R, Q.R, MCG.R, IFGT.R, VA.R, STG.R, and MTG (ROI 23, 24). Some group-specific patterns have also been detected: the ROIs at SFGM.R for preadolescent group; the ROIs at IFGT.R and MTG.L for early adolescent group; the ROIs at SFG.L, MCG.R, and VA.R for middle adolescent group; the ROIs at MCG.L (ROI: 5, 8), and STG.R for late adolescent group; and the ROIs at AMY.L and SFG.R for postadolescent group.

#### Robustness of the findings regarding sample size.

**Figure 7.**
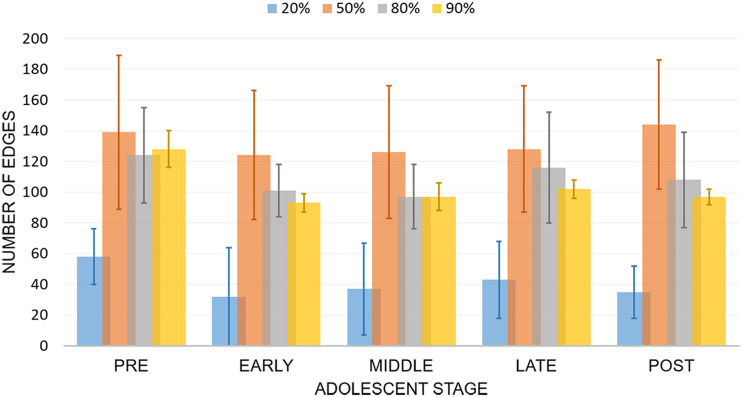
The robustness of BiLiNGAM with various percentages (20%, 50%, 80%, 90%) of samples (*N* = 855) from the PNC dataset. Data were mean number of edges ± standard deviation.

We examined the robustness of our proposed method and assessed the effect of sample size on the robustness. We randomly drew *m* percent of *N* participants (*m* ∈ {20%, 50%, 80%, 90%}, *N* = 855) from the PNC dataset sample without replacement and with the proportion of the five adolescent groups. We then applied the BiLiNGAM method to the subsamples. For each sample size, we repeated the procedure 10 times. In Figure 7, we showed the mean number of edges detected for each adolescent group under various sample sizes. From preadolescence to postadolescence, the trajectory of each sample size remains similar. However, we found that limited edges were identified with small sample size (*m* = 20%). As the sample size increases, the identified number of edges for each group remains steady and the variance decreases significantly.

#### Comparison with other dFC methods.

To prove the effectiveness and reliability of the proposed method, we compared the dFC estimated by BiLiNGAM, *ψ*-LiNGAM (Zhang, Zhang, et al., 2020) and the PC-algorithm (Spirtes et al., 2000) using the fMRI data from the PNC study. We calculated the Jaccard index as in Zhang, Padmanabhan, et al. (2019) to quantify the graph similarities among different adolescent groups. The Jaccard index is defined as the ratio of the intersection between two sets of edges divided by their union, ranging from 0 (no overlap) to 1 (full overlap). As shown in Table 3 (the columns of among groups), the BiLiGAM method has significantly reinforced the group similarities (specially when compared with *ψ*-LiNGAM).

**Table 3.** A comparison of the graph similarities using the mean Jaccard index (with standard deviation)

Among methods		Among groups	
v.s. BiLiNGAM	Jaccard index	Method	Jaccard index
PC	0.133 (0.014)	PC	0.120 (0.006)
*ψ*-LiNGAM	0.230 (0.140)	*ψ*-LiNGAM	0.050 (0.039)
		BiLiNGAM	0.151 (0.023)

We then compared the method-wise similarities (see Table 3, columns of among methods). Fisher’s exact test was applied to examine the independence of the dFC’s estimated by the three methods. The *p* values were all lower than 10^−25^, indicating that the estimated dFC from the three methods are significantly correlated. The comparison with PC algorithm shows lower Jaccard index. This discrepancy can be attributed to the limitations of the PC algorithm in accurately identifying the orientation of edges. As a result, the PC algorithm often produces graphs with numerous undirected edges, which may lead to differences in the estimated graphs compared to our proposed method. The *ψ*-LiNGAM method exhibits higher similarities with BiLiNGAM. However, these similarities are less consistent across groups as indicated by the variance of Jaccard index. This inconsistency is due to sample size limitations, as demonstrated in the simulation studies.

## DISCUSSION

In this paper, we propose a multiple DAG estimation method for non-Gaussian data, BiLiNGAM, and further apply it to the study of brain development. The main contributions of our work can be summarized in three primary points. First, from a mathematical perspective, we relaxed the assumption of common ordering from the joint LiNGAM (Shimizu, 2012) and made it possible to estimate larger graphs. Second, our proposed method integrated undirected and directed graphs, by incorporating the undirected graph estimation as prior information into the direct LiNGAM model for better DAG construction. We use the undirected graphs to mitigate the irrelevant information for better casual inferences, in addition to optimizing convergence and computation. Third, the simulation results show that BiLiNGAM maintains a stable and accurate performance over various settings. In particular, the proposed BiLiNGAM is superior for high-dimensional cases. Finally, the analysis of brain’s emotion circuit development revealed the trajectory of directed brain circuitry during emotion identification tasks over various adolescent groups. We identified several significant intra- and intermodular networks that change over developmental stage, and pinpointed emotion-related hubs as well as various group-specific patterns.

### Intramodular Development

We found a developmentally stable intramodular activation anchored in the default mode (DMN), subcortical (SCN), and cerebellum (CERE) networks. The default mode network is important for mentalizing and inferring emotional states of others (Blakemore, 2008); subcortical regions have a pivotal role in cognitive, affective, and social functions in humans (Koshiyama et al., 2018); the cerebellum contributes prominently in processing emotional facial expression (Ferrucci et al., 2012). The intramodular activities of CERE increased significantly in the postadolescent group, which may emerge in late puberty (Tiemeier et al., 2010). The significance of intramodular activities of the SCN appeared from preadolescence to middle adolescence, which has been proven to be an important developmental period for subcortical brain maturation (Dennison et al., 2013).

### Intermodular Development

As age increases, more intermodular connectivity emerges during emotion-related processing. Starting from early adolescence, interconnections start to build among VAN, CON, SN, and SSN. Specifically, SSN emerges with significant interconnections that receives information from other functional networks after middle adolescence. VAN plays an important role in conveying information in late adolescent group, and SN is crucial for receiving information in postadolescent group. Two stable directed influences from CON to SSN, and from DMN to MRN become established after the late adolescent period. CON facilitates the maintenance of task-relevant goals and the incorporation of error information to adjust behaviors (Cocchi, Zalesky, Fornito, & Mattingley, 2013) and SSN (including somatosensory cortex, motor regions, and supplementary motor areas) is involved in performing and coordinating motor-related tasks like finger tapping. Gehringer et al. (2019) proposed that maturation of the somatosensory system during adolescence contributes to improved motor control. They further discovered that altered attenuation of the somatosensory cortical oscillations might be central to the underdeveloped somatosensory processing and motor performance characteristics in adolescents. Our results agree with their conclusion and provide additional explanations. Besides, Sestieri, Corbetta, Romani, and Shulman (2011) confirmed that responses in DMN regions peaked sooner than non-DMN regions during memory retrieval, and the parietal regions of DMN directly supported memory retrieval.

### Emotion-Related Hubs

Most identified hubs are located in the right hemisphere, which are dominant in the perception of facial expression and important for processing primary emotions (Alfano & Cimino, 2008). Particularly, the hubs at PG, MCG, PQ, and MTG play central roles in socioemotional processing. The precentral gyrus (PG) of the somatosensory cortex is related to the recognition of facial and vocal expressions of emotion and a main effect of emotional valence on brain activity has been found in the PG.R (Seo et al., 2014). A previous study (Shackman et al., 2011) verified that the mid-cingulate gyrus (MCG) is a hub linking incoming affective information with brain regions involved in goal-directed behavior, and we further discovered that it is also a hub for distributing affective information. Precuneus (PQ) activation has been implicated in emotional and memory-loaded processes. The current study suggests that the PQ may play a direct role in the regulation of amygdala reactivity to emotional stimuli (Ferri, Schmidt, Hajcak, & Canli, 2016), which explains its prominence as a out-hub location. Studies of emotional face recognition (Haxby, Hoffman, & Gobbini, 2002; Sabatinelli et al., 2011) identified the middle temporal gyrus (MTG) as a primary neural substrate for suprathreshold processing of the emotional expression of faces, which is consistent with our result of MTG as a central node to pass out information.

### Developmental Hub Structures and Group-Specific Hub Patterns

The majority of networks during development fluctuate, except for the steady increase of the in-hub activities at the PQ.R and ACG.R. Group-specific patterns have also been identified: the in-hub at SMA.L and out-hub at SFGM.R for preadolescence; the out-hubs at IFGT.R and MTG.L for early adolescence; the out-hubs at SFG.L, MCG.R, and VA.R for middle adolescence; the out-hubs at MCG.L and STG.R for late adolescence; the out-hubs at AMY.L and SFG.R for postadolescence. Some of our results have been previously supported in the literature. The role of developmental centers at PG, PQ, ACG, LING, and PHIG remains consistent with our previous study of brain connectivity development in adolescence (Zhang, Padmanabhan, et al., 2019). In this study, we further pinpoint their specific functions in the emotion circuit through directed graphical models. Another study of brain development from adolescence to adulthood (Kundu et al., 2018) also brought attention to age-related changes in the PQ. In Simmonds, Hallquist, and Luna (2017), fluctuating trajectories in the MCG during adolescence were discovered.

Our findings provide a directed functional connectivity template of emotion processing in the developing brain, thereby shedding light on the understanding of brain networks underlying emotion processing behaviors.

## SUPPORTING INFORMATION

Supporting information for this article is available at https://doi.org/10.1162/netn_a_00384.

## AUTHOR CONTRIBUTIONS

Aiying Zhang: Conceptualization; Formal analysis; Investigation; Methodology; Validation; Visualization; Writing – original draft; Writing – review & editing. Gemeng Zhang: Conceptualization; Investigation; Methodology; Writing – review & editing. Biao Cai: Conceptualization; Visualization; Writing – review & editing. Tony W. Wilson: Funding acquisition; Supervision; Writing – review & editing. Vince D. Calhoun: Funding acquisition; Supervision; Writing – review & editing. Julia Stephen: Funding acquisition; Supervision; Writing – review & editing. Yu-Ping Wang: Data curation; Funding acquisition; Resources; Supervision; Writing – review & editing.

## FUNDING INFORMATION

Tony W. Wilson, National Institute of Mental Health (https://dx.doi.org/10.13039/100000025), Award ID: R01MH121101. Yu-Ping Wang, National Institute of Mental Health (https://dx.doi.org/10.13039/100000025), Award ID: R01MH104680. Yu-Ping Wang, National Institute of Mental Health (https://dx.doi.org/10.13039/100000025), Award ID: R01MH107354. Vince D. Calhoun, National Institute of Mental Health (https://dx.doi.org/10.13039/100000025), Award ID: R01MH103220. Vince D. Calhoun, National Institute of General Medical Sciences (https://dx.doi.org/10.13039/100000057), Award ID: P20GM109068. Tony W. Wilson, National Institute of General Medical Sciences (https://dx.doi.org/10.13039/100000057), Award ID: P20GM144641. Vince D. Calhoun, National Institute of Biomedical Imaging and Bioengineering (https://dx.doi.org/10.13039/100000070), Award ID: R01EB020407. Yu-Ping Wang, National Institute of Mental Health (https://dx.doi.org/10.13039/100000025), Award ID: R56MH124925.

## Supplementary Material



## TECHNICAL TERMS

Emotion circuit: A network of brain regions involved in the generation, processing, and regulation of emotions.
Functional magnetic resonance imaging (fMRI): A neuroimaging technique that measures brain activity by detecting changes associated with blood flow.
Functional connectivity: Statistical dependencies between the time series of activation patterns in different brain regions.
Directed acyclic graph (DAG): A DAG is a probabilistic graphical model that represents a set of variables and their conditional dependencies via the directed edges.
Linear non-Gaussian acyclic model (LiNGAM): A statistical modeling technique to estimate a DAG for non-Gaussian distributed data.
Functional networks: Sets of brain regions that exhibit synchronized or coordinated activity during specific cognitive tasks or while the brain is at rest.
Bayesian prior: In Bayesian statistics, a prior probability distribution, often simply called a prior, represents the state of knowledge or belief about the parameters of interest before observing any data.
