# Supplementary material for: A Bayesian incorporated linear non-Gaussian acyclic model for multiple directed graph estimation to study brain emotion circuit development in adolescence
Source: Netw Neurosci. 2024 Oct 1;8(3):791–807. doi: 10.1162/netn_a_00384 (PMC11349030; doi:10.1162/netn_a_00384)
Supplement: Supplementary file 1 [file netn-8-3-791-s001.pdf]

A Simulation Studies

In the section, our goal is to demonstrate the superiority of the proposed BiLiNGAM methods comprehensively through a series of simulation studies. In Appendix A, we first illustrated the advantages of incorporation prior information for single DAG estimations. To be more specific, we have compared the ψ-LiNGAM method [1] which used partial correlation as prior, with the ICA-LiNGAM [2] and direct-LiNGAM [3] methods (without prior), as well as other two well-known DAG estimation methods: the PC algorithm [4], and the greedy equivalent search (GES) [5]. In Appendix B, we further explained the necessity of joint estimation for multiple groups that are related but distinct.

We assessed the performance of each method through the true positive rate (TPR), false discovery rate (FDR), and structural hamming distance (SHD) [6]. SHD is a commonly used metric based on the number of operations needed to transform the estimated DAG into the true graph [7]. In simple terms, SHD counts the total number of edge insertions, deletions or flips during the transformation. TPR and FDR are two typical measures of a binary classification. Let us define an experiment from P positive instances and N negative instances for some conditions. In our case, the positive instance represents a directed edge from one node to the other. The four outcomes are summarized in Table 1. The definitions of TPR and FDR are given as follows:

$$\text{TPR} = \frac{\text{TP}}{\text{TP} + \text{FN}}, \quad \text{FDR} = \frac{\text{FP}}{\text{FP} + \text{TP}}.$$

Table 1: Outcomes of a binary decision

	Actual positive (P)	Actual negative (N)
Predicted positive	True positive (TP)	False positive (FP)
Predicted negative	False negative (FN)	True negative (TN)

A.1 Single DAG estimation with existing methods

We compared 4 existing methods, which were the well-known PC algorithm [4] and GES [5] method, two LiNGAM methods: ICA-LiNGAM [2] and the ψ-LiNGAM method [1]. Among them, the LiNGAM methods were designed for non-Gaussian distributed data and the ψ-LiNGAM incorporated prior information for estimations. We simulated the random DAG G through the R package *pcalg* with the edge probability $d/(p-1)$, where d is an edge degree parameter and p is the total number of variables. Given G, we assigned uniformly random weights to the edges to obtain the weighted adjacency matrix $\mathbf{B}$: $b_{ij} \sim \text{Unif}(-0.8, -0.3) \cup (0.3, 0.8)$, if $b_{ij} \in E$, otherwise $b_{ij} = 0$. Given $\mathbf{B}$, we generated $\mathbf{x} = \mathbf{B}^T\mathbf{x} + \boldsymbol{\epsilon} \in \mathbb{R}^p$ from the chi-squared (Chisq) noise with degree of freedom 1 and zero mean, i.e., $\epsilon_i \sim \chi_1^2 - 1$, $i = 1, 2, ..., p$. We then sampled the random vectors $\mathbf{x} \in R^{n \times p}$ with $n = 500$, $p = 50, 100, 200$ and the degree parameter $d = 1, 2, 4$ based on the models. For each scenario, we simulated 10 datasets independently. All these algorithms can be implemented through the R package *pcalg*. We set the significance level $\alpha = 0.05$ (with FDR correction) for all 4 methods to obtain the estimated DAGs.

The simulation results are shown in Figure A. As we can see, the ψ-LiNGAM has significantly improved performance over direct-LiNGAM. The ψ-LiNGAM has the highest TPR while maintaining a low range of FDR under each setting. Although the SHD grows with the variable size p, the increasing slope of the ψ-LiNGAM is the lowest as well as the SHD value. Overall, the ψ-LiNGAM has outperformed the other methods under each setting, especially under large variable number and/or low degree parameter setting. For PC algorithm and GES, the low TPR and high FDR are caused by their poor direction identification. To be more specific, both PC and GES can only estimate the completed partially directed acyclic graph (CPDAG), which contains both undirected and directed edges [4, 5]. For the undirected edges in PC and GES, we treat them as bi-directional to calculate the metrics. The high SHD value of GES is because the GES method tends to identify more false positive edges. Although LiNGAM has a decent performance compared to PC and GES, the false discovery rate has increased

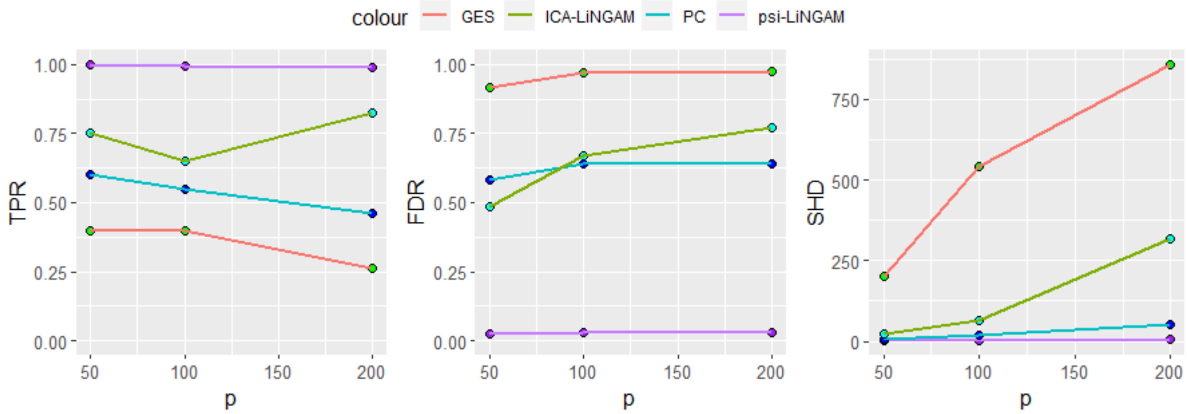

(a) d=1

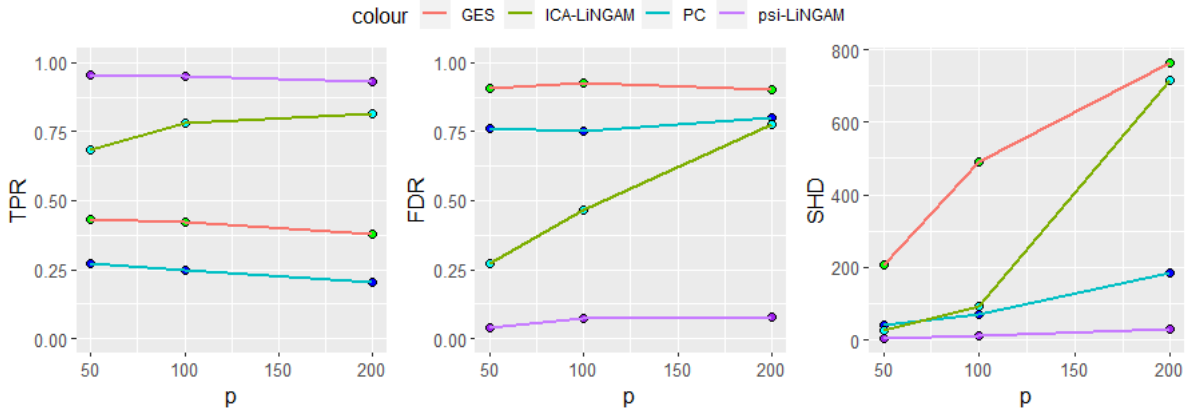

(b) d=2

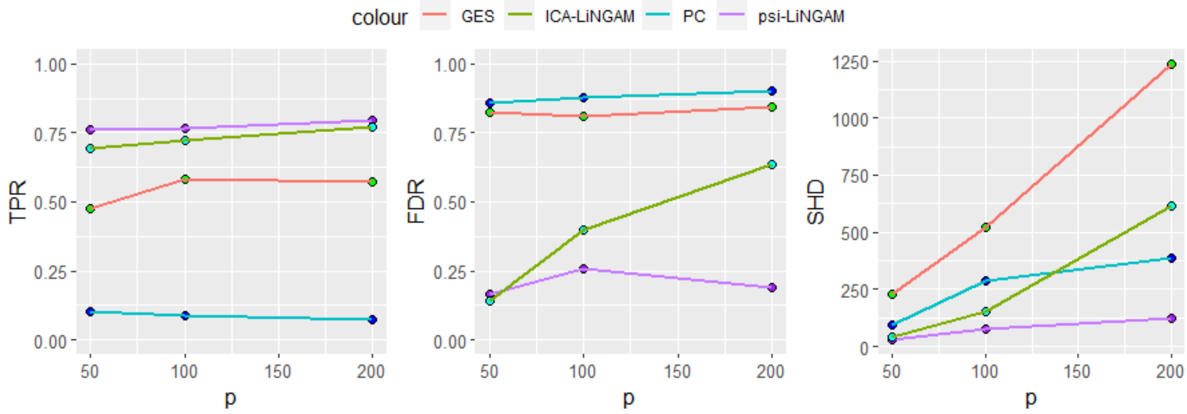

(c) d=4

Figure A: Simulation results with chi-squared noise setting, which represent the average performance in terms of TPR, FDR and SHD under various variable ($p = 50, 100, 150$) and degree parameter ($d = 1, 2, 4$) settings with $n = 500$.

significantly as the variable size increases. This is because the original LiNGAM method needs a large number of samples in the relevant dimension to converge.

A.2 Multiple DAG estimation comparisons

We analyzed the performance of estimating of K different but related DAGs. We considered 4 methods, which were the proposed the BiLiNGAM method, the ψ-LiNGAM, the ICA-LiNGAM, and the PC. We selected the better performed methods between the PC and GES based on the results from Appendix A. We set the number of nodes $p = 200$ for all experiments and considered two scenarios:

1. We fixed the total number of observations $N = 750$ and varied the number of groups $K \in \{3, 5, 10\}$.

2. We fixed the number of groups $K = 5$ and varied the total number of observations $N \in \{375, 750, 1250\}$.

For each group, we set the number of samples equally, i.e. $n_1 = n_2 = \cdots = n_K = n = N/K$. The random DAG G can be simulated through the R package *pcalg* and density of the graph is controlled by the edge probability $d/(p-1)$, where d is an edge degree parameter with values $\{1, 2, 5\}$. The true DAGs generation procedure is illustrated as follows. We first used the *pcalg* to generate G^1. Given G^1, we assigned uniformly random weights to the edges to obtain the weighted adjacency matrix $\mathbf{B}^1 = (b^1_{ij})$: $b^1_{ij} \sim \text{Unif}(-0.8, -0.3) \cup (0.3, 0.8)$, if there is an edge $i \to j$, otherwise $b^1_{ij} = 0$. For G^k, $k = 2, 3, \ldots, K$, we followed the same random edge deleting-adding procedure in a sequential manner. We randomly removed 5% edges in G^{k-1}, $k = 2, ..., 5$, by setting the corresponding non-zero elements in $\mathbf{B}^k$ to be 0, and then added 5% edges at random by giving them values drawn from the uniform distribution $U[0.3, 0.5]$ to obtain $\mathbf{B}^k$. Given $\mathbf{B}^k$'s, we generated $\mathbf{X}^k = (\mathbf{B}^k)^T \mathbf{X}^k + \boldsymbol{\epsilon}^k \in R^p$ with $\boldsymbol{\epsilon}^k$ from Chi-squared (Chisq) noise with degree of freedom 1 and zero mean, i.e. $\epsilon^k_i \sim \chi^2_1 - 1$, $i, = 1, 2, \ldots, p$, $k = 1, 2, \ldots, K$.

For each case of each scenario, 10 datasets were simulated independently. Note that the ICA-LiNGAM cannot be applied to high-dimensional cases. From Fig. B and Figure C, we can see as N fixed and K increased or K fixed and N decreased, the overall performances of the methods decreased as expected. On the contrary, the advantages of the BiLiNGAM has become more apparent, as it keeps a high TPR while maintain a steady FDR and SHD. Overall, BiLiNGAM has maintained a stable and accurate performance over various settings. Particularly, under high dimensional cases, the performance of BiLiNGAM is superior. When the sample size is adequate (i.e. $n > p$), BiLiNGAM performs at least as good as ψ-LiNGAM. Since both of the variations are related to the group sample size n. Based on our simulation settings, we were also able to present the results with fixed n: 1. $n = 75$, $K \in \{5, 10\}$; 2. $n = 250$, $K \in \{3, 5\}$. As shown in Figure E, we observe that the BiLiNGAM method is sensitive to group sample size n but not the number of groups K.

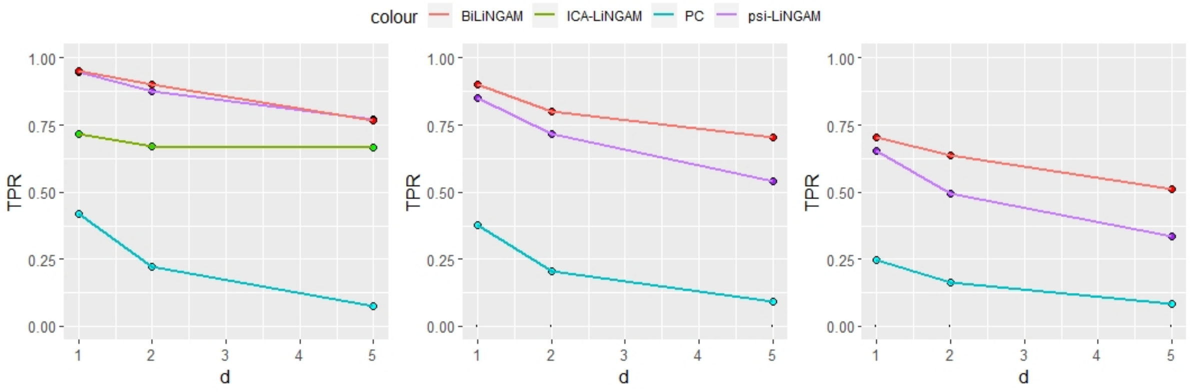

(a) TPR

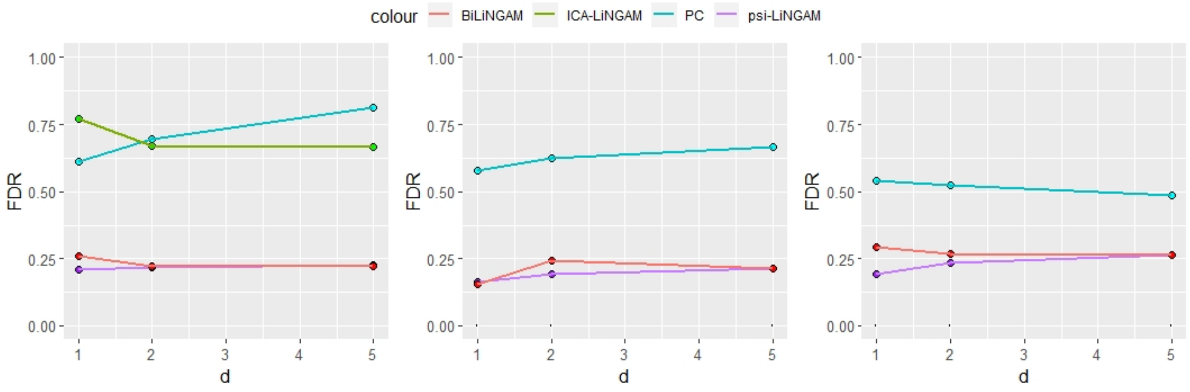

(b) FDR

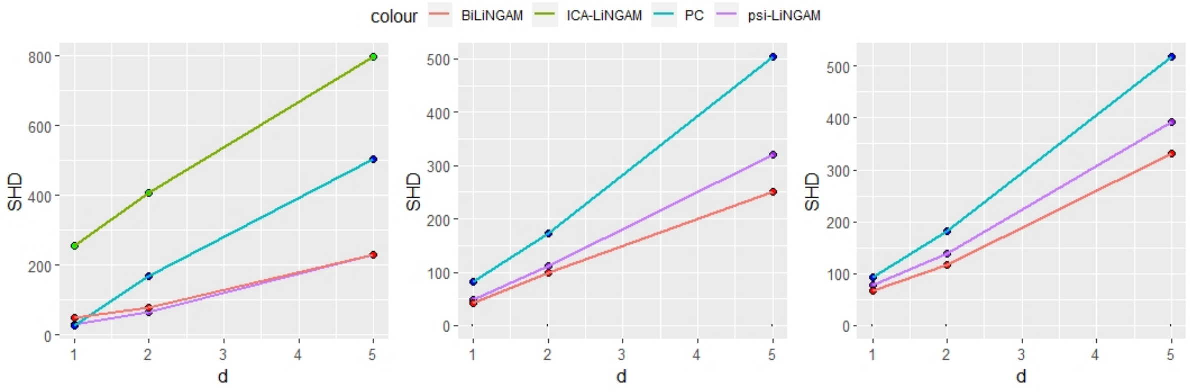

(c) SHD

Figure B: Simulation results for scenario 1, which from left to right represent the average performance for $K = (3, 5, 10)$, respectively.

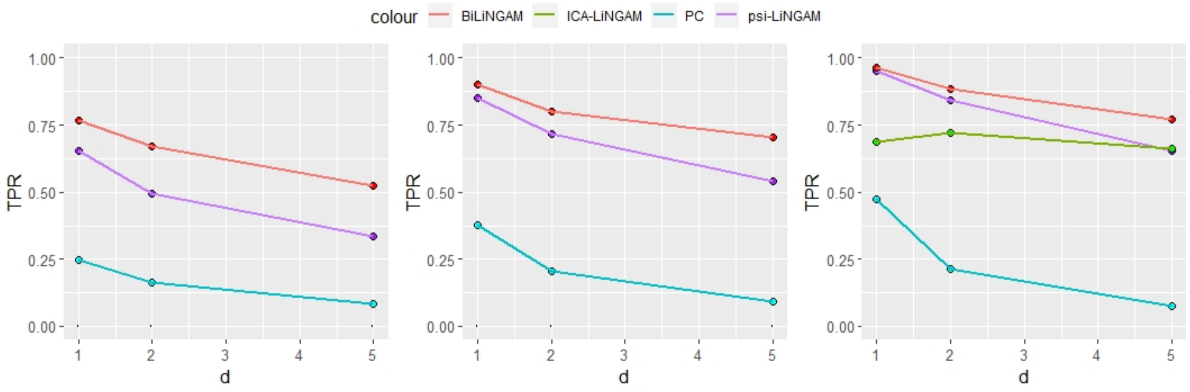

(a) TPR

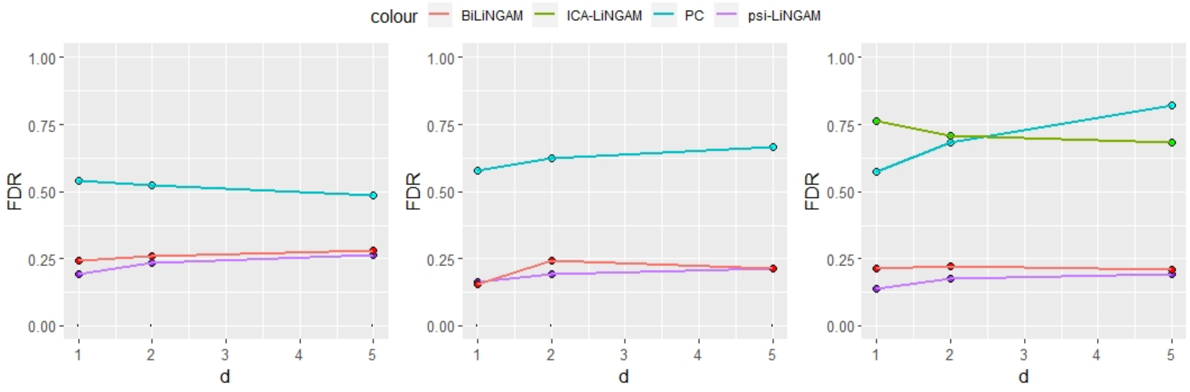

(b) FDR

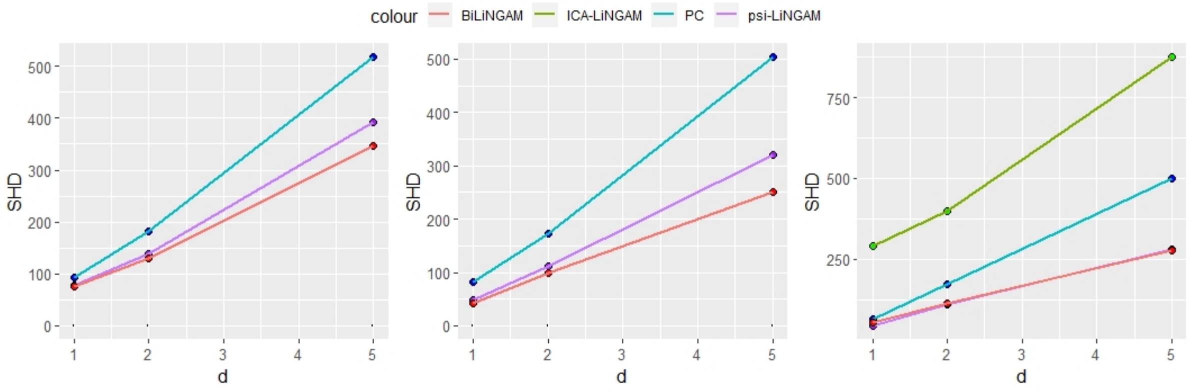

(c) SHD

Figure C: Simulation results for scenario 2, which from left to right represent the average performance for $n = (75, 150, 250)$, respectively.

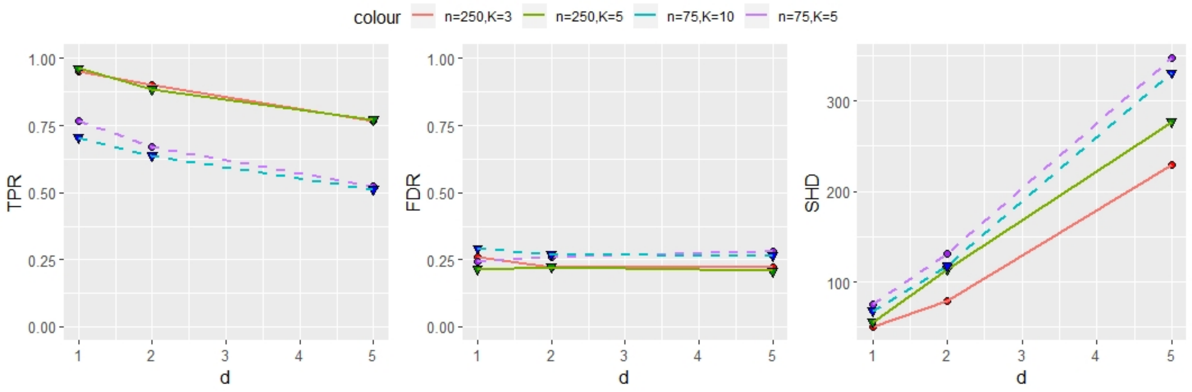

Figure D: Comparisons of the average performance of BiLiNGAM with fixed n while varying K.

B Additional results on the PNC studies

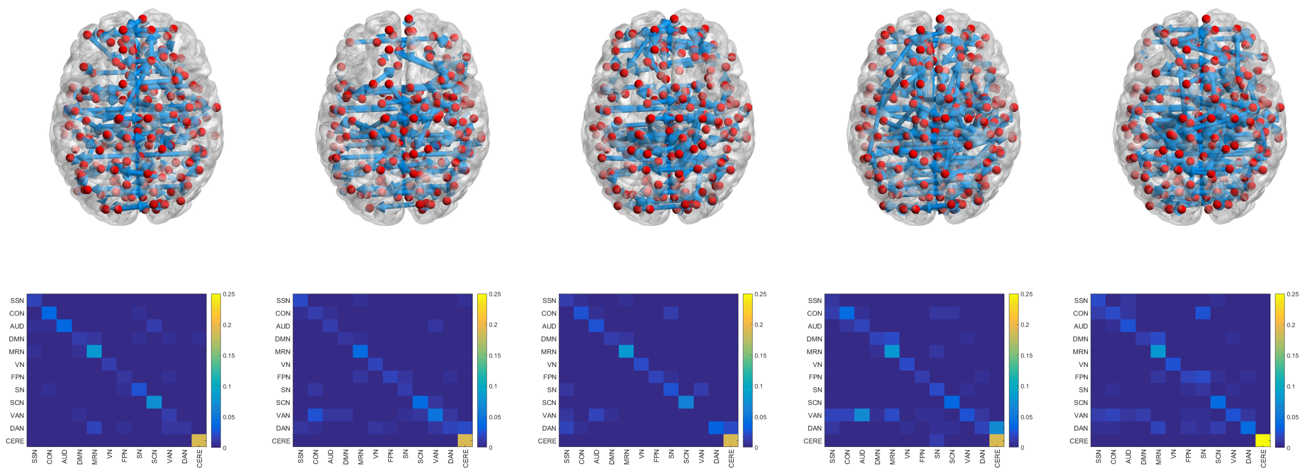

Figure E: Directed brain connectivity development from pre-adolescence (left) to post-adolescence (right). The top row shows the axial views of emotion-related node-level directed FC, where the arrows indicate the causal flow. Only the nodes that have connections are visualized. The bottom row presents the heatmaps of the mean edge degrees, module-wise.

Table 2: Anatomical location, functional network module and MNI coordinates of the identified in-hub ROIs.

ROI	MNI (X, Y, Z)	Module	AAL	Abbrev.
1	20, -29, 60	SSN	Precentral Gyrus (R)	PG.R
2	8, -48, 31	DMN	Mid-cingulate gyrus (R)	MCG.R
3	-2, -35, 31	MRN	Posterior cingulate gyurs (L)	PCG.L
4	4, -48, 51	MRN	Precuneus (R)	PQ.R
5	-10, 11, 67	VAN	Supplementary motor area (L)	SMA.L
6	36, 22, 3	SN	Insula (R)	INS.R
7	10, 22, 27	SN	Anterior cingulate gyrus (R)	ACG.R
8	12, -17, 8	SCN	Thalamus (R)	THA.R

* The ROI index corresponds to the row order from Fig. **??** (b).

Table 3: Anatomical location, functional network module and MNI coordinates of the identified out-hub ROIs.

ROI	MNI (X, Y, Z)	Module	AAL	Abbrev.
1	-14 -18 40	SSN	Amygdala (L)	AMY.L
2	29 -17 71	SSN	Precentral gyrus (R)	PG.R
3	-40 -19 54	SSN	Precentral gyrus (L)	PG.L
4	19 -8 64	CON	Superior frontal gyrus (R)	SFG.R
5	-10 -2 42	CON	Mid-cingulate gyrus (L)	MCG.L
6	-13 -40 1	DMN	Precuneus (L)	PQ.L
7	15 -63 26	DMN	Precuneus (R)	PQ.R
8	-2 -37 44	DMN	Mid-cingulate gyrus (L)	MCG.L
9	-10 55 39	DMN	Superior frontal gyrus (L)	SFG.L
10	-20 45 39	DMN	Superior frontal gyrus (L)	SFG.L
11	13 30 59	DMN	Superior frontal gyrus, medial (R)	SFGM.R
12	-26 -40 -8	DMN	Parahippocampus (L)	PHIP.L
13	18 -47 -10	VN	Lingual gyrus (R)	LG.R
14	-15 -72 -8	VN	Lingual gyrus (L)	LG.L
15	6 -72 24	VN	Cuneus (R)	Q.R
16	11 -39 50	SN	Mid-cingulate gyrus (R)	MCG.R
17	48 22 10	SN	Inferior frontal gyrus, triangular (R)	IFGT.R
18	37 32 -2	SN	Inferior frontal gyrus, orbital (R)	IFGO.R
19	26 50 27	SN	Middle frontal gyrus (R)	MFG.R
20	9 -4 6	SCN	Ventral Anterior Nucleus (R)	VA.R
21	52 -33 8	VAN	Superior temporal gyrus (R)	STG.R
22	51 -29 -4	VAN	Middle temporal gyrus (R)	MTG.R
23	-52 -63 5	DAN	Middle temporal gyrus (L)	MTG.L
24	46 -59 4	DAN	Middle temporal gyrus (R)	MTG.R
25	29 -5 54	DAN	Precentral gyrus (R)	PG.R

* The ROI index corresponds to the row order from Fig. **??** (c).

References

[1] Aiying Zhang, Gemeng Zhang, Biao Cai, Wenxing Hu, Li Xiao, Tony W Wilson, Julia M Stephen, Vince D Calhoun, and Yu-Ping Wang. Causal inference of brain connectivity from fmri with ψ-learning incorporated

linear non-gaussian acyclic model (ψ-lingam). *arXiv preprint arXiv:2006.09536*, 2020.

[2] S. Shimizu, P.O. Hoyer, A. Hyvärinen, and A. Kerminen. A linear non-gaussian acyclic model for causal discovery. *Journal of Machine Learning Research*, 7:2003–2030, 2006.

[3] Shohei Shimizu, Takanori Inazumi, Yasuhiro Sogawa, Aapo Hyvärinen, Yoshinobu Kawahara, Takashi Washio, Patrik O Hoyer, and Kenneth Bollen. Directlingam: A direct method for learning a linear non-gaussian structural equation model. *The Journal of Machine Learning Research*, 12:1225–1248, 2011.

[4] P. Spirtes, C.N. Glymour, R. Scheines, D. Heckerman, C. Meek, G. Cooper, and T. Richardson. *Causation, prediction, and search.* MIT press, 2000.

[5] D.M. Chickering. Optimal structure identification with greedy search. *Journal of machine learning research*, 3:507–554, 2002.

[6] I. Tsamardinos, L.E. Brown, and C.F. Aliferis. The max-min hill-climbing bayesian network structure learning algorithm. *Machine learning*, 65:31–78, 2006.

[7] M. Kalisch and P. Bühlmann. Estimating high-dimensional directed acyclic graphs with the pc-algorithm. *Journal of Machine Learning Research*, 8:613–636, 2007.
